# Method-centered digital communities on protocols.io for fast-paced scientific innovation

**DOI:** 10.12688/f1000research.9453.2

**Published:** 2017-06-29

**Authors:** Lori Kindler, Alexei Stoliartchouk, Leonid Teytelman, Bonnie L. Hurwitz

**Affiliations:** 1 Department of Agricultural and Biosystems Engineering, University of Arizona, Tucson, AZ, 85721, USA; 2 protocols.io, Berkeley, CA, 94704, USA

**Keywords:** forum, virtual community, protocols, metagenomics, bioinformatics, virus, phage, bacteriophage

## Abstract

The Internet has enabled online social interaction for scientists beyond physical meetings and conferences. Yet despite these innovations in communication, dissemination of methods is often relegated to just academic publishing. Further, these methods remain static, with subsequent advances published elsewhere and unlinked. For communities undergoing fast-paced innovation, researchers need new capabilities to share, obtain feedback, and publish methods at the forefront of scientific development. For example, a renaissance in virology is now underway given the new metagenomic methods to sequence viral DNA directly from an environment. Metagenomics makes it possible to “see” natural viral communities that could not be previously studied through culturing methods. Yet, the knowledge of specialized techniques for the production and analysis of viral metagenomes remains in a subset of labs.  This problem is common to any community using and developing emerging technologies and techniques. We developed new capabilities to create virtual communities in protocols.io, an open access platform, for disseminating protocols and knowledge at the forefront of scientific development. To demonstrate these capabilities, we present a virology community forum called VERVENet. These new features allow virology researchers to share protocols and their annotations and optimizations, connect with the broader virtual community to share knowledge, job postings, conference announcements through a common online forum, and discover the current literature through personalized recommendations to promote discussion of cutting edge research. Virtual communities in protocols.io enhance a researcher’s ability to: discuss and share protocols, connect with fellow community members, and learn about new and innovative research in the field.  The web-based software for developing virtual communities is free to use on protocols.io. Data are available through public APIs at protocols.io.

## Introduction

The Internet has enabled online social interaction for scientists beyond physical meetings and conferences. Twitter, Facebook, and ResearchGate ^1– 3^ provide valuable online forums that many researchers use to share knowledge. At the same time, academic publishing remains time consuming and inefficient for communicating methodology. Protocols are often relegated to supplementary information, if shared at all. There is no good mechanism for easily discussing, troubleshooting, and improving published or unpublished techniques.

This need is even more apparent in emerging fields such as viral ecology where laboratory, field, and bioinformatics methods are being actively developed ^4^. For example, new metagenomic techniques to sequence viral DNA directly from environmental samples has led to rapid advances in both molecular and bioinformatic protocols ^5^. These protocols, however, are highly specialized and generally used in a few highly proficient labs because: (i) viral metagenomes (viromes) are difficult to produce due to low quantities of DNA and refined isolation and purification methods, (ii) the vast majority of viral sequences are unknown (usually >90% ^6^) complicating bioinformatics analyses, and (iii) newly emerging comparative and functional metagenomic analyses exist but require on-going community refinement and development. 

Given the experimental nature of methods, the virology community has expressed a need to foster discussions about these protocols towards improved methodologies and increasing connectivity and collaboration among researchers ^7^. The challenge is to develop a method-centered collaborative platform that recapitulates the functionality of a scientific meeting - a digital community for connecting with fellow researchers to share and discover state-of-the-art knowledge.

Here we describe new capabilities in protocols.io ( http://www.protocols.io), an open access platform, to create virtual communities to disseminate protocols and knowledge at the forefront of scientific development. To demonstrate these capabilities, we describe a viral ecology community forum called VERVENet ( https://www.protocols.io/groups/verve-net) that strives to increase connectivity and knowledge dissemination in viral ecology research at all levels from undergraduates to accomplished viral ecologists. These new community features enhance a researcher’s ability to discuss and share protocols, connect with fellow community members, and learn about new and innovative research in the field. The web-based software for developing virtual communities is free for use on protocols.io, and further described here.

## Protocols.io: a platform to enable methods discussion and dissemination

Protocols.io is a free service for industry and academic scientists to share or maintain private protocols for research ^8^. The driving force behind software development is to provide a mechanism for scientists to share improvements and corrections to protocols, so that others are not continuously re-discovering knowledge that scientists have not had the time or wear-with-all to publish. Protocols.io provides a free, up-to-date, crowd-sourced protocol repository called protocols.io ( http://www.protocols.io) for the life science community. This software is available as a web-based platform or smart phone App ^9, 10^ to enable mobile solutions for research and bench-work. Per best practices in mobile computing, these Apps offer extensive options and control of push notifications. In fall 2014, protocols.io offered a well-developed platform for users to share molecular methods, however no capabilities were in place to share methods among groups, or bioinformatics methods. To this end, the viral ecology community teamed up with protocols.io to create new group capabilities, develop bioinformatics protocols, and enhance discussion forums for news, methods, and literature.

**Introducing VERVENet: the Viral Ecology Research and Virtual Exchange Network:** The Viral Ecology Research and Virtual Exchange Network (VERVENet), is a collaboration between the University of Arizona and protocols.io, to deliver an online forum for the virology community. To enable this forum, new group functionality was built into protocols.io to promote scientific communication and collaboration. Specifically, group features were developed on top of existing capabilities to share molecular methods in order to ( *i*) share protocols and their annotations and optimizations, (ii) fuel connectivity among viral ecology researchers for sharing data sets, knowledge, job postings, conference announcements through a common online forum called VERVENet and ( *iii*) facilitate literature discovery through personalized recommendations to promote discussion on cutting edge viral ecology research. Through developing these interconnected resources in protocols.io for virtual communities, we developed a “go-to” site for viral ecology research ^11^. Moreover, these tools are broadly useful to any community or individual lab for promoting scientific inquiry, reproduction of results, dissemination of protocols and re-use. Specifically, new forums can be created in a matter of minutes to enable connectivity among groups of any size, with tools described here under use cases. The VERVE Net forum is a place to discuss newly emerging methods in viral ecology for any kind of data such as: ‘omics or image datasets. Yet, while images, videos and tables can be added to protocols/steps to enhance the description of methods, the protocols.io platform is not a data storage site.

## Methods

**Creating a user profile in protocols.io:** Users can view protocols and all public content anonymously, but to interact with the platform, registration is necessary. Registration is quick as only e-mail and password are required to create an account; however, users are encouraged to create profiles containing their name, website, affiliation, and research interests. Others can search and find a user based on name or keywords. Moreover, user profiles are attached to any material on protocols.io that the user posts publically. User profiles also contain a field for ORCID ^12^, so that researchers can tie their profile back to a common identifier and highlight their work in the field. Researchers can also include a biography that describes how they got into the field and what intrigues them. Thus, profiles allow users to add in their own content, rather than simply browse existing content.

**Figure 1.**
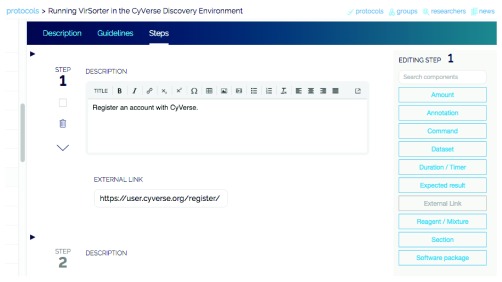
Entering a protocol in protocols.io. Protocols are entered by providing a broad description, information about authors, any prior materials or background required, and detailed step-by-step methods to implement the protocol. Protocols can remain private to an individual or group, or released to the public.

**Adding protocols in protocols.io:** After registration, new protocols can be entered ( Figure 1). By default, all protocols are private and can be shared with individual collaborators or any of the groups. The protocols are structured with tabs for the “steps”, “description”, ‘guidelines’, and “comments”. When entering the steps, a list of components that can be added to the steps is located on the far right and allows a clear detailing of wetlab or computational portions of the method. Related steps of the protocol can also be easily grouped together into “sections” such as ‘preparation,’ ‘DNA extraction,’ and ‘analysis’, *etc*. Steps may be entered one by one by typing into the text box or by pasting steps from another file, facilitating import of existing protocols. For each step annotations can be added to make notes on specific steps. Once complete, the protocol can be “run” in a step-by-step format.

Once a protocol has been created, there are several options for sharing it with collaborators or a group. To make the protocol publicly viewable, one will need to click the ‘publish’ button. A protocol can be reassigned to another individual with a protocols.io account. For ongoing development and changes to adding and using protocols, see tutorials ( http://www.protocols.io/help) in protocols.io ^13^.

**Developing groups in protocols.io**: To create a group, one must have an account and be logged in. For example, here we describe the VERVE Net group, however it is possible to create any group. To create a group, users can click on their personal icon in the upper right hand corner and select “+ new group.” They will be prompted to enter a group name, image, description of the group, research interests, external website address, physical location of the group and an affiliation. The user will also decide if the group is open to anyone, by invitation only, or open to membership requests. In addition, the user can choose if the group is visible to others or private. Users are able to invite members into their group and control the privileges of their members. Moreover, as the owner of a group, the user is able to invite other subgroups, such as in the VERVE example individual labs are subgroups.

**Finding protocols:** Protocols on protocols.io can be “tagged” to allow users to quickly find protocols or collections of protocols in a particular area of interest. Users can also find protocols or other content using the global search at the top of each page that allows users to search within the entire forum, or specific sections of the forum.

**Providing feedback on protocols:** protocols.io offers three methods for feedback directly from users: twitter, email to protocols developers (info@protocols.io), and through a feedback forum where users and developers alike can respond. These comments are then used to fuel future development. Further, protocols.io recently initiated an ambassadors program where power users (usually graduate students or postdocs) that are directly connected to diverse communities provide feedback from a user-perspective. Thus, future development is guided by community input from these sources.

## Use case: VERVE Net: Virus Ecology Research and Virtual Exchange Network

**Molecular and bioinformatics protocols:** Often, detailed “tricks of the trade” associated with lab, field, and bioinformatics protocols are not well-described in publications, and at best are stashed in supplemental materials. Practical information associated with running these protocols under varied conditions cannot be curated, documented, or discussed among students, postdocs, technicians, and faculty working in virology. Moreover, knowledge on when to use a particular version of a given protocol is not easily captured. Protocols.io provides a flexible mechanism wherein protocols can be documented in a stepwise fashion to easily pivot between molecular and bioinformatics methodologies, link to useful websites or code in Github ^14^, or reference manuals or original source materials for protocols, as exemplified in the VERVENet forum.

The user entering the protocol may not necessarily be the author of the original method. However, by providing links to the primary work, users can attribute credit to the original author while at the same time adding their own updates to the method either while they enter it, or at a later time. Further, other users have the capability to add notes and warnings to existing protocols in protocols.io. This functionality includes a mechanism to email the protocol author for protocol troubleshooting. Corrections and updates made by the protocol authors and users automatically trigger notifications e-mailed to researchers who use that protocol. Lastly, users can “fork” or copy existing protocols for further refinement or alternate uses while still maintaining links back to the original for credit and reference. As such, the protocol is a living document for the community to reuse and continually refine.

For publication, authors have the option to enter detailed methods into protocols.io, issue a digital object identifier (DOI ^15^), and link to the protocols.io record from the Methods section. This practice is now being encouraged in journal submissions and by funding agencies.

**Protocol collections:** Because protocols are often used in conjunction with other protocols, protocols.io has the capability to link protocols into user-defined workflows. This is particularly important for publications that may use a collection of varied protocols (field, lab, and bioinformatics) that are derived from many sources (protocols from the user or other users). In providing a collection of protocols associated with a publication, the authors enable their work to be replicated, easy to follow, and transparent to other members of the community in a way that can be referenced and cited. For example, a collection of protocols derived from a recent publication on the human skin double stranded DNA skin virome is available in VERVENet ^16, 17^. Thus, collections provide a mechanism for furthering open-science efforts.

Protocol collections also provide a mechanism to “learn by example” for early career scientists or those branching into a new area of scientific inquiry. In particular, detailed protocols associated with a toolkit or workshop, where multi-media options such as slides, video, or links to virtual machines with example datasets and code can be included ^18, 19^. This is particularly important for bioinformatics protocols that often include multiple programs and steps in an analysis for a given publication. Further, individual tools may have a collection of protocols that describe specific use-cases, example datasets, and varied options that they may wish to convey to their users.

**Figure 2.**
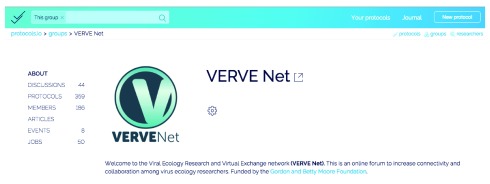
The VERVENet group in protocols.io. Groups in protocols.io display information about the group objectives, members, subgroups, the group library and literature recommendations, group discussions, news, jobs, and events. Groups have the capacity to control access, from making groups and content public and allowing anyone to join, to restricted content and invitation only membership. VERVENet is an example of a public forum for virology.

**Groups and sharing:** Individual members can form groups, where the owner has the ability to choose the level of accessibility for fellow members. The groups can share literature recommendations, discussions, protocols, news, events, and job opportunities ( Figure 2). Subgroups can form under the umbrella of a larger group with a common interest. This subgroup/supergroup relationship allows smaller group activities to be shared with a larger virtual community with common interests. In the case of VERVENet, this supergroup links the broader research in virology with the subgroups of individual labs and more specific research interests such as plant viruses.

**Literature recommendations:** Each of the groups includes a literature recommendation system. This algorithm provides personalized publication recommendations based on a library from a user or group. This algorithm is used to develop “libraries” for viral ecology user groups, that will continually recommend new publications based on growing reading lists from individual users that are part of the group. This functionality allows virologists to make their reading lists public therefore helping new scientists joining the field in their topic area. The libraries from “sub-groups” also fuel the shared public reading list within the VERVENet group, therefore creating enhanced fluidity and cross-posted content between the groups.

**Live online discussion forum:** Each of the groups in protocols.io contains a live on-line discussion. Discussions can be generated directly on the discussion tab, or are cross-posted from discussions on specific protocols, news, or literature. Each of the discussions can reference outside websites, manuals, or online resources. This discussion forum enables users to discuss tips and tricks for specific protocols, review reagents linked to particular protocols, and reference outside resources that were not included in the original protocol.

Protocols.io also includes “journal-club” capabilities to enable on-line discussions of published research by researchers and authors. Other unique features in protocols.io include: career advice forum including a panel of mentors ^20^ and a “ *behind the article*” essay forum ^21^. These communication forums allow researchers to share their stories about how papers, protocols, or research efforts came about, that are both interesting to the community and informative for early career scientists.

**Platform infrastructure and interoperability:** Computers, tablets, and smart phones are becoming fundamental tools for scientists today. Furthermore, social networking and shared cyberinfrastructures are offering powerful new mechanisms to connect communities and science from across the world. Protocols.io leverages these powerful new tools and software capabilities to provide an online forum for viral ecology research to connect and share knowledge and resources. All components of protocols.io and the VERVENet forum are mobile-friendly and interoperable for use on diverse devices in the lab, on the desktop, or on the go.

**Content and adoption:** The VERVENet group currently contains 365 live protocols, 212 news articles, and 59 job opportunities. There is an event calendar that contains workshops and conferences specific to virology through the fall of 2016. We have 231 members and 22 subgroups. Examples of subgroups include: Plant Virus Ecology Network which originally formed in 2007 ^22^, the Chlorovirus Group, ECOGEO ^23^, and 18 individual labs. The International Society for Viruses of Microorganisms has listed VERVE Net on their website ^24^ as a resource.

## Discussion and conclusions

The primary goal of new group functionality in protocols.io is to provide a robust web-application for sharing up to date protocols, literature, and community features (news, jobs, discussions). This work is exemplified in VERVE Net, a virtual community forum for virology. Fundamental to this goal is the ability for researchers to establish groups based on similar interests and share knowledge, without *apriori* knowledge of key members in a given field. 

We have designed an infrastructure that has multiple entry points for establishing relationships among users ranging from self-proclaimed groups or areas of interest, to options to join groups maintained by others in an area of interest to the user fueled by related protocols or reading lists. Moreover, news feeds about funding opportunities, job postings, or collaborative research opportunities can be fine-tuned according to interest. These connections will allow the forum to evolve naturally given rapidly developing trends and new protocols. Protocols.io is open access and is both, free to read and free to publish. The revenue and sustainability model is based on the sale of data services to reagent vendors (most popular protocols, protocol improvements, and reagent-protocol links). Protocols.io also charges fees for private non-academic groups.

Protocols.io is a central resource to connect, collaborate, share and innovate within virtual communities. The VERVENet forum demonstrates how this new group functionality allows researchers to promote scientific inquiry, reproduction of results, and dissemination and optimization of both molecular and bioinformatics protocols, as a virtual community. 

## Data and software availability

Protocols.io and the VERVENet commuity forum are committed to open access for data content and interoperability. To that end, the content in protocols is available through an Application Programming Interface (API) for advanced data mining and no registration is required to view protocols, comments or annotations. All public protocols are archived with CLOCKSS for long-term digital preservation ^25^. Users will also be able to access public protocols.io mirrored at the Center for Open Science.
