# Supplementary material for: Method-centered digital communities on protocols.io for fast-paced scientific innovation
Source: F1000Res. 2017 Jun 29;5:2271. Originally published 2016 Sep 8. [Version 2] doi: 10.12688/f1000research.9453.2 (PMC5506532; doi:10.12688/f1000research.9453.2)
Supplement: Peer review history

# Peer review: Referee response for version 2

**DOI:** 10.5256/f1000research.12719.r23915

**Published:** 2017-07-11

**Authors:** David D. Dunigan, Irina Agarkova

**Affiliations:** 1 Nebraska Center for Virology, Department of Plant Pathology, University of Nebraska- Lincoln, Bethesda, MD, USA; 2 University of Nebraska-Lincoln, Lincoln, NE, USA

We have read this submission. We believe that we have an appropriate level of expertise to confirm that it is of an acceptable scientific standard.

# Peer review: Referee response for version 1

**DOI:** 10.5256/f1000research.10182.r16953

**Published:** 2016-10-18

**Authors:** Lawrence Patrick Kane

**Affiliations:** 1 Department of Immunology, University of Pittsburg, Pittsburgh, PA, USA

This well-written report describes the creation of a new platform for facilitating collaborative work among and between research groups. This platform takes advantage of the existing protocols.io site and App, and so is focused on methodological sharing, although other types of information are also covered. This approach fits well with the recent emphasis (from NIH and others) to address the issue of reproducibility in the biomedical literature. Of course, the ultimate utility of this (or any) platform will depend on what those in the field make it.

 One item that the authors may wish to clarify is the protocols.io business model, which is touched upon near the end of this piece, but is likely not familiar to many readers. This is not meant to impugn the data services model, but just to ensure its transparency.

I have read this submission. I believe that I have an appropriate level of expertise to confirm that it is of an acceptable scientific standard.

# Response

**Published:** 2017-01-03

**Authors:** Bonnie Hurwitz

**Affiliations:** University of Arizona, USA

Thank you for reviewing our manuscript. We have updated the text to clarify the business model for protocols.io per your suggestion.

# Peer review: Referee response for version 1

**DOI:** 10.5256/f1000research.10182.r16177

**Published:** 2016-09-16

**Authors:** David D. Dunigan, Irina Agarkova

**Affiliations:** 1 Nebraska Center for Virology, Department of Plant Pathology, University of Nebraska- Lincoln, Bethesda, MD, USA; 2 University of Nebraska-Lincoln, Lincoln, NE, USA

The paper describes a relatively new on-line forum referred to as VERVENet that was developed with the private company protocols.io.  VERVENet seeks to provide a virtual gathering place for viral ecologists where information on experimental protocols, literature, upcoming conferences and job listings are centralized and made available, openly. The paper describes most of these features and provides some examples to help encourage the reader to explore VERVE Net online.

 There are some concerns that may need further clarification in this publication relating to 1) navigation, 2) types of data being supported, and 3) intellectual property rights.

  

1. At a practical level, VERVENet is still in its infancy and one would expect it to continue to develop in response to the community input. Thus, the concerns we have with navigating in VERVENet at this time will likely be corrected/ adjusted with continued use and input by users. The authors should address how they intend to respond to the intended community when suggestions are proposed. Who has the “authority” to make the changes and how will those changes be made available? Given the potential (and presumed intended) for a highly dynamic forum, will there be an archive of information maintained?
2. It is not quite clear from the manuscript how navigation by particular topics works. Is it possible to find a specific protocol without having to scroll through all entries, as well as to find easily a collection of protocols related to a particular field, e.g,. nucleic acid isolation and manipulation; proteomics; bioinformatics, etc? If these functions exist in the current version of VERVENet, it would help to describe this more fully. 
3. The authors indicate that VERVENet was initiated, in part, due to “…a renaissance in virology..given the new metagenomic methods...” Although it is true that there is a large influx of metagenomic data in recent years, DNA sequence data is not the only type of data of interest to viral ecologists. The authors should discuss what types of data are being supported. For example, how are image data sets being handled, given the relatively large file size? How will VERVENet adapt as other types of ‘omics’ data become common within the viral ecology community? Clearly, DNA sequence is not the only data of interest, RNA sequence is equally important to virologists, so the authors may wish to indicate the range of data intended for VERVENet.
4. The authors have taken on the responsibility of creating an online forum with an emphasis on methods. These methods are created by individuals and generally made available to anyone whether they register with protocols.io or not, at varying levels depending on the authors’ decisions. It is not clear who holds the intellectual property rights at that point. The authors should be transparent about the rights of the protocols.io contributing authors, where the legal authority begins and ends, and how contributors can both share and protect their intellectual property should they choose. Another concern that should be addressed is what is the fate of VERVENet should protocols.io change ownership. Are there contingency plans in the company statutes that would ensure that groups like VERVENet be supported? What happens if they are not supported? Who has legal ownership the information? Also, given that the current funding is through the Gordon and Betty Moore Foundation, what happens to the content of VERVENet should that funding be removed or reduced? These things need some clarification. 
5. The title of the paper does not give a full reflection of the contents of the paper.
6. There are several statements in the manuscript where the authors tend to use emphatic language that is not easily supporter. For example, the first sentence of the Abstract infers that social interactions of scientists ONLY occurs at physical meetings and conferences. This statement is not only inaccurate, it undermines the credibility of the information that follows. The authors should re-read their paper and consider how some of their statements may be perceived. There are several examples.

We have read this submission. We believe that we have an appropriate level of expertise to confirm that it is of an acceptable scientific standard, however we have significant reservations, as outlined above.

# Response

**Published:** 2017-05-26

**Authors:** Bonnie Hurwitz

**Affiliations:** University of Arizona, USA

Thank you for your time in reviewing our manuscript.  Please see our comments to your suggested revisions below:

**Comment 1**: At a practical level, VERVENet is still in its infancy and one would expect it to continue to develop in response to the community input. Thus, the concerns we have with navigating in VERVENet at this time will likely be corrected/ adjusted with continued use and input by users. The authors should address how they intend to respond to the intended community when suggestions are proposed. Who has the “authority” to make the changes and how will those changes be made available? Given the potential (and presumed intended) for a highly dynamic forum, will there be an archive of information maintained?

**Response 1**: In terms of the software, protocols.io offers three methods for feedback directly from users: twitter, email to protocols developers (info@protocols.io), and through a feedback forum where users and developers alike can respond.  These comments are then used to fuel future development.  Further, protocols.io recently initiated an ambassadors program where power users (usually graduate students or postdocs) that are directly connected to diverse communities provide feedback from a user-perspective.  VERVE Net is one such community and currently has an ambassador to further develop the community.  Thus, future development is guided by community input from these sources.  We have amended the manuscript to clarify this important point.

Information from the forum is maintained through the CLOCKKS digital archiving project ( https://www.clockss.org/clockss/Home) as described in the manuscript under “Data and software availability”.

**Comment 2:** It is not quite clear from the manuscript how navigation by particular topics works. Is it possible to find a specific protocol without having to scroll through all entries, as well as to find easily a collection of protocols related to a particular field, e.g,. nucleic acid isolation and manipulation; proteomics; bioinformatics, etc? If these functions exist in the current version of VERVENet, it would help to describe this more fully.

**Response 2: **Protocols on protocols.io can be “tagged” to allow users to quickly find protocols or collections of protocols in a particular area of interest.  Users can also find protocols or other content using the global search at the top of each page that allows users to search within the entire forum, or specific sections of the forum.  We have updated the text to clarify the search features.

**Comment 3**: The authors indicate that VERVENet was initiated, in part, due to “…a renaissance in virology..given the new metagenomic methods...” Although it is true that there is a large influx of metagenomic data in recent years, DNA sequence data is not the only type of data of interest to viral ecologists. The authors should discuss what types of data are being supported. For example, how are image data sets being handled, given the relatively large file size? How will VERVENet adapt as other types of ‘omics’ data become common within the viral ecology community? Clearly, DNA sequence is not the only data of interest, RNA sequence is equally important to virologists, so the authors may wish to indicate the range of data intended for VERVENet.

**Response 3**: Indeed, VERVENet is not just a forum to discuss ‘omics datasets but is inclusive of all types of data on studying viruses.  The forum is meant to be a place to discuss newly emerging methods in viral ecology that includes but is not limited to ‘omics datasets. Also, while images, videos and tables can be added to protocols/steps, the protocols.io platform is not a data storage site like dropbox, GitHub, figshare, datadryad or CyVerse.  To clarify, we added several sentences in the introduction.

**Comment 4**: The authors have taken on the responsibility of creating an online forum with an emphasis on methods. These methods are created by individuals and generally made available to anyone whether they register with protocols.io or not, at varying levels depending on the authors’ decisions. It is not clear who holds the intellectual property rights at that point. The authors should be transparent about the rights of the protocols.io contributing authors, where the legal authority begins and ends, and how contributors can both share and protect their intellectual property should they choose. Another concern that should be addressed is what is the fate of VERVENet should protocols.io change ownership. Are there contingency plans in the company statutes that would ensure that groups like VERVENet be supported? What happens if they are not supported? Who has legal ownership the information? Also, given that the current funding is through the Gordon and Betty Moore Foundation, what happens to the content of VERVENet should that funding be removed or reduced? These things need some clarification.

**Response 4**: All public content on protocols.io is open access and is clearly labeled as CC-BY at the footer of each page. Moreover, the Terms of Service (https://www.protocols.io/terms#tos1) are explicit: "We claim no intellectual property rights over the material you provide to the Sites/Apps. Your profile and materials uploaded remain yours. However, by publishing your protocols to be viewed publicly, you agree to allow others to view your Content. By setting your protocols to be viewed publicly, you agree to allow others to view and fork your protocols."

Content generated by our users belongs to the users and is licensed under the Creative Commons Attribution License (CC-BY). As discussed above, the archiving via CLOCKSS and the mirroring with COS are meant to ensure long-term digital preservation of all knowledge on protocols.io, even in the case of bankruptcy or change of control.

VERVENet does not rely on continued funding from GBMF. The support for this and other groups comes from the revenue streams at protocols.io. Ensuring long-term sustainability, independent of grants from funding agencies was an important component of the original VERVE grant application.

**Comment 5**: The title of the paper does not give a full reflection of the contents of the paper.

**Response 5**: The title reflects the general application of developing digital communities in protocols.io for which VERVENet is an example.  We kept the title more general to reflect the broad utility of the forum. While VERVENet was the first group on protocols.io and the grant for it enabled the creation of the functionality described here, it is meant for wide use and has already growth to more than 250 public and private groups and communities such as PROT-G, Open Plant, MinION, and others.

**Comment 6**: There are several statements in the manuscript where the authors tend to use emphatic language that is not easily supporter. For example, the first sentence of the Abstract infers that social interactions of scientists ONLY occurs at physical meetings and conferences. This statement is not only inaccurate, it undermines the credibility of the information that follows. The authors should re-read their paper and consider how some of their statements may be perceived. There are several examples.

**Response 6**: Thank you for pointing this out.  Social interactions at conferences was only meant to be an example of the many interactions scientists have daily.  We updated the manuscript to omit/rephrase any emphatic language per your suggestion.
